# Racial differences in associations between adverse childhood experiences and physical, mental, and behavioral health

**DOI:** 10.1016/j.ssmph.2023.101524

**Published:** 2023-10-08

**Authors:** Tracy Lam-Hine, Corinne A. Riddell, Patrick T. Bradshaw, Michael Omi, Amani M. Allen

**Affiliations:** a Stanford University School of Medicine, Division of Epidemiology & Population Health, 1701 Page Mill Road, Palo Alto, CA, USA; b University of California Berkeley School of Public Health, Division of Biostatistics, 2121 Berkeley Way West, Berkeley, CA, USA; c University of California Berkeley School of Public Health, Division of Epidemiology, 2121 Berkeley Way West, Berkeley, CA, USA; d University of California Berkeley Department of Ethnic Studies, 506 Social Science Building, Berkeley, CA, USA; e University of California Berkeley School of Public Health, Division of Community Health Sciences, 2121 Berkeley Way West, Berkeley, CA, USA

**Keywords:** Adverse childhood experiences, Racial groups, Asthma, Anxiety

## Abstract

**Purpose:**

Adverse childhood experiences (ACEs) are associated with poor adulthood health. Multiracial people have elevated mean ACEs scores and risk of several outcomes. We aimed to determine whether this group should be targeted for prevention efforts.

**Methods:**

We analyzed three waves (1994–2009) of the National Longitudinal Study of Adolescent to Adult Health (n = 12,372) in 2023, estimating associations between four or more ACEs and physical (metabolic syndrome, hypertension, asthma), mental (anxiety, depression), and behavioral (suicidal ideation, drug use) outcomes. We estimated adjusted risk ratios for each outcome in modified Poisson models interacting race and ACEs. We used the interaction contrast to estimate race-specific excess cases per 1000 relative to Multiracial participants.

**Results:**

Excess case estimates of asthma were smaller for White (−123 cases, 95% CI: −251, −4), Black (−141, 95% CI: −285, −6), and Asian (−169, 95% CI: −334, −7) participants compared to Multiracial participants. Black (−100, 95% CI: −189, −10), Asian (−163, 95% CI: −247, −79) and Indigenous (−144, 95% CI: −252, −42) participants had fewer excess cases of and weaker relative scale association with anxiety compared to Multiracial participants.

**Conclusions:**

Adjusted associations with asthma and anxiety appear stronger for Multiracial people. Existing ACEs prevention strategies should be tailored to support Multiracial youth and families.

## Highlights

• Multiracial people have elevated mean ACEs (adverse childhood experience) scores.
• They also face risk of poor health in some outcomes, including anxiety and asthma.
• These outcomes' associations with ACEs are strongest for the Multiracial group.
• ACEs may contribute disproportionately to morbidity in this population.

## Abbreviations

Add Health: The National Longitudinal Study of Adolescent to Adult Health
ACE: Adverse childhood experience; potentially traumatic events that occur in childhood
AI/NA: American Indian/Native American
CI: confidence interval
MetS: Metabolic syndrome; a cluster of conditions occurring together associated with increased risk of heart disease, stroke, and type 2 diabetes
RR: risk ratio

## 1 Introduction

Adverse childhood experiences (ACEs), also referred to as childhood or early life adversity, are traumatic events during childhood and adolescence that are linked to poor health in adulthood, including five (heart disease, cancer, respiratory diseases, diabetes, and suicide) of the ten leading causes of death in the United States (Centers for Disease Control and Prevention; Petruccelli et al., 2019; Kalmakis & Chandler, 2015). Over 60% of the US population reports at least one ACE, with up to a third reporting household emotional abuse, parental divorce, or substance abuse, specifically (Felitti et al., 1998; Merrick et al., 2018). While children exposed to ACEs may show immediate signs of distress, the health impacts of ACEs may manifest over time, as exposure to trauma early in life predisposes individuals to stressful situations later in life through what has been termed the stress proliferation chain (Hertzman & Power, 2003; Jones et al., 2018; Manyema et al., 2018; Nurius et al., 2015). Trauma is theorized to embed biologically along at least three distinct pathways to influence health: (1) triggering of the biological stress response and resulting impacts on the neuroimmune-endocrine axis (Kelly-Irving et al., 2013; Stewart, 2006), (2) epigenetic expression, (CDC, 2020; Labonté et al., 2012; Moore et al., 2013) and (3) changes in health behaviors due to adaptation or coping with severe or long-term stress (Su et al., 2015; Vig et al., 2020; Wekerle et al., 2020; Wiss & Brewerton, 2020).

Racial disparities in ACE scores are well-documented, with Multiracial and American Indian/Native American (AI/NA) populations reporting the highest mean ACE score of any racial group (Cronholm et al., 2015; Giano et al., 2020; Kenney & Singh, 2016; Merrick et al., 2018; Skewes & Blume, 2019). In addition to higher ACE scores, Multiracial people report high prevalence of health conditions traditionally linked with ACEs, and poor health in several other conditions. Some studies suggest Multiracial adolescents have poor sleep quality and behavioral and mental health (Choi et al., 2006; Doyle, 2007; Goings et al., 2018; Goodhines et al., 2020; Pang, 2015; Straka et al., 2019; Udry et al., 2003). Multiracial adults experience the highest prevalence of heart diseases, asthma, obesity, hopeless feelings, and serious psychological distress of any racial group (National Center for Health Statistics, 2018a; National Center for Health Statistics, 2018b; National Center for Health Statistics, 2018c; National Center for Health Statistics, 2018d), and are significantly more likely to be living in poverty, uninsured, and in poor physical health than monoracial White people (Subica et al., 2017; Summary Health Statistics, 2018). Despite these patterns and the growing size of the Multiracial population, investigators frequently continue to reclassify Multiracial participants into a catchall “other” racial category, existing monoracial categories, or exclude them completely, effectively masking disparities affecting this group (Charmaraman et al., 2014; Facente et al., 2022).

To our knowledge, only two ACEs studies (Hall et al., 2020; LaBrenz et al., 2020) have estimated associations specifically for Multiracial populations. Hall et al.’s (Hall et al., 2020) study of asthma found that Multiracial participants had the highest mean ACE score and asthma prevalence of any racial group, but no differences in race-specific interaction odds ratios from the reference group (Asians). LaBrenz et al. (LaBrenz et al., 2020) modeled days of poor mental and physical health, and estimated additive interactions between race and ACE scores. Interaction terms in the mental health model were positive for the Black, Asian, Pacific Islander, Multiracial and “Other” racial groups compared to Whites. For poor physical health days, interaction effect estimates were close to zero and less precise. One small study of monoracial White and Black adolescents in the US South found a stronger dose-response relationship between ACE score and depressive symptoms scores for Black compared to White participants (Youssef et al., 2017). Other studies examining monoracial adolescents (Stinson et al., 2021) and adults (Assini-et al., 2022; Lee & Chen, 2017) have generally found no moderation of health outcomes by race. These findings suggest that the strength of some ACE-health outcome relationships may vary across racial groups, with the direction depending on the referent racial group.

In this study, we leverage a nationally representative sample of young adults to determine if the association between ACEs and a range of common physical, behavioral, and mental health outcomes are stronger for Multiracial people, and by extension, if prevention efforts should target this group.

## 2 Materials and methods

### 2.1 Study sample

The National Longitudinal Study of Adolescent to Adult Health (Add Health) is a longitudinal, nationally representative study following over 20,000 individuals enrolled in grades 7–12 in 1994-95 through four waves of follow-up (1996, 2001-02, 2008-09, 2016-18) (Harris, 2013). Eighty middle and high schools were selected non-randomly for size, type, grade range, setting, demographics, and geographic location. Students were sampled randomly without stratification from these schools' enrollment rosters and invited to complete an at-home interview during Wave 1 (n = 20,745) which asked questions about the adolescent's demographics, family background, social networks, home and school environments, and health behaviors. Wave 3 (n = 15,197) collected additional life experience and medication information. Wave 4 (n = 15,701) was conducted when participants were in their late twenties and included measurements of participants' metabolic and cardiovascular function. We did not use Wave 2 data as it did not contain outcome relevant for this study. Details about the Add Health study, including analysis of the impact of non-response at Wave 4, are available elsewhere (Brownstein et al., 2018; Chen, 2014; Harris, 2013).

We categorized participants into five racial groups: White alone, Black alone, Asian alone, American Indian/Native American (AI/NA) alone, (hereafter White, Black, Asian, or AI/NA) or Multiracial. Waves 1 and 3 asked participants to self-identify their race; we used Wave 3 race unless a participant identified as Multiracial at Wave 1 but not Wave 3, in which case we classified them as Multiracial. We excluded Hispanic/Latino participants because race and Hispanic ethnicity are assessed independently in Add Health, making it impossible to fully enumerate Hispanic/Latino participants that also identify as Multiracial. This is because in contrast to the current federal racial/ethnic categorization schema, some (but not all) Hispanic/Latino individuals regard this label as a racial identity (Taylor et al., 2012; Udry et al., 2003).

### 2.2 Measures

Because differential exposure to ACEs may be linked to racial disparities in health through the stress pathway, we investigated physical, mental, and behavioral outcomes (groupings described below) for which (1) Multiracial Americans experience relatively poor health outcomes, and (2) are associated with ACEs and overactivation of the stress pathway (Chen & Miller, 2007; Cuevas et al., 2017; Exley et al., 2015; National Heart et al., 2020; Williams & Neighbors, 2001). We measured outcomes once for each individual in either Wave 3 (ages 18–26) or 4 (24–32) based on data availability. Appendix A contains details on construction of outcomes and covariates.

• *Physical health: metabolic syndrome (MetS), hypertension, and asthma.* We assessed MetS using clinically-relevant cutoffs for eight biomarkers across five categories: (1) hypertension (systolic blood pressure ≥130 mmHg, diastolic blood pressure ≥80 mmHg), (Whelton et al., 2017) self-reported ever diagnosed with hypertension, or self-reported current hypertension medication use), (2) waist circumference (>102 cm for males, >80 cm for females), (3) triglycerides (top 3 deciles for males, top 2 for females), (4) high-density lipoprotein (bottom 2 deciles for males, bottom 3 for females), and (5) diabetes (glycated hemoglobin >5.7%, self-reported anti-diabetic medication use, or self-reported diabetes diagnosis) (Bohr et al., 2016). We categorized individuals with values exceeding cutoffs in three or more of the five categories as having MetS (Bohr et al., 2016; Colen et al., 2020; Gaydosh et al., 2018; Martin et al., 2019; Miller et al., 2020). Hypertension coding when considering it as a separate outcome mirrored that used for the MetS measure. We coded participants as having asthma if they indicated a previous diagnosis (Harris et al., 2006).
• *Mental health: depression and anxiety.* We classified participants as having depression if they indicated using prescription depression medications in the last year or having a previous depression diagnosis, and as having anxiety if they indicated a previous anxiety diagnosis.
• *Behavioral health: suicidal ideation and drug use.* We coded participants as having suicidal ideation if they indicated seriously thinking about committing suicide in the last year, and using drugs if they indicated ever using cocaine, crystal meth, heroin, other illegal drugs other than marijuana, or ever misusing prescription drugs.

The original ACEs questionnaire included ten questions each covering a domain of adverse experiences: emotional, physical, and sexual abuse, emotional and physical neglect, parental separation or divorce, mother treated violently, household substance abuse, household mental illness, and incarceration of household member. (About the CDC-Kaiser ACE Study) Researchers constructing ACEs scores using Add Health data have used varying sets of questions about events occurring before age 18 to approximate the original ACEs questionnaire (Brumley et al., 2017; Easterlin et al., 2019; Lee et al., 2020; LeTendre & Reed, 2017; Otero, 2021). We used a modified version of the widest set of questions available, some of which were answered by participants’ parents in Wave 1, and others retrospectively by participants during Waves 3 or 4. We dichotomized and summed responses to create a summary score from zero to ten. We categorized participants reporting four or more ACEs as exposed, as a previous meta-analysis found elevated risk above this level for all studied negative physical, mental, and behavioral health outcomes (Hughes et al., 2017). We conducted sensitivity analyses using the summary score instead of a dichotomized exposure. Questions and response categories used are shown in Appendix B.

We identified confounders using a directed acyclic graph. We adjusted our models for age, sex, parental education, household size-adjusted income, parental support, and neighborhood disadvantage score as confounders. We drew age and sex from Wave 4 to match timing of collected biomarkers. The Wave 4 questionnaire asked respondents to report their gender but only provided “male” and “female” as response options; we thus consider this variable to represent biological sex. Following previous Add Health analyses, we categorized parental education as the higher of either parent's Wave 1 parental education after categorization into a five-level ordinal variable (less than high school, completed vocational school, or GED; high school diploma; some college; college graduate or greater) (Goodman et al., 2003). We calculated household size-adjusted income as total reported pre-tax household income divided by the square root of total reported household members at Wave 1. (Organization for Economic Cooperation and Development) We coded parental support as the mean of Likert-scale responses (very much, quite a bit, some, a little, none at all) to five Wave 1 questions about participants' relationship and communication with their parents (Chen & Harris, 2019; Sieving et al., 2000). We constructed a crude neighborhood disadvantage score by averaging five census tract-level proportions from participants' Wave 1 residence: (1) percent households with incomes below the federal poverty level, (2) percent households receiving public assistance, (3) civilian unemployment rate, (4) percent persons 25 years or older with no high school diploma or equivalency, and (5) percent female-headed households (Martin et al., 2019; Ross & Mirowsky, 2001).

### 2.3 Statistical analyses

For each outcome, we specified a modified Poisson model to estimate risk ratios (Zou, 2004). We visually assessed assumptions of linearity between exposure and log-transformed risk of outcomes for each racial group by comparing observed data with linear model-generated smooth plots. All models interacted ACEs and race to estimate subgroup-specific associations. Because our aim was to understand if associations are stronger for Multiracial people, we specified Multiracial participants as the reference group in all interactions. We also conducted sensitivity analyses with Whites as the referent group to compare findings with previous studies which have used this approach.

We used complex survey weights corresponding to a cross-sectional multi-wave analysis to produce nationally representative estimates using the “survey” package in R (Chen, 2014; Lumley, 2004). Because 55% of observations were missing data on an ACE component, 21% on a covariate, and 40% on an outcome (frequencies presented in Appendix C), we used multiple imputation including all outcomes in imputations models, and pooled results across 20 imputed datasets (Harel et al., 2018). We imputed data using the “mi” and “mitools” packages in R (Lumley, 2019; Su et al., 2011). We summed and dichotomized ACE scores in regression analyses after imputation of missing ACE component variables.

We assessed interactions on the relative scale by estimating subgroup specific risk ratios (RRs), and on the absolute scale by estimating excess cases per 1000. We calculate excess cases by recovering the interaction contrast (IC) from the interaction contrast ratio (or relative excess risk due to interaction), and then converting the IC to excess cases (VanderWeele & Knol, 2014). Further details on calculation of excess cases is available in Appendix D. We assessed differences between group RRs and excess case estimates by comparing magnitude of associations and coverage of 95% confidence intervals (CI) around estimates.

The University of California, Berkeley Office for Protection of Human Subjects determined that this study did not meet the threshold definition of human subjects research; we conducted analyses in 2023.

## 3 Results

Post-hoc analysis led us to exclude individuals who identified as “Other” race alone due to small sample size (n = 23) and overly wide CIs. Table 1 shows key characteristics (unweighted counts and weighted statistics pooled from imputations) in the overall study sample. Sample size in models with self-reported outcome data was 12,372. Biomarkers can be affected by pregnancy, thus we excluded 445 pregnant participants from the MetS and hypertension models. In the overall study sample, there were 7742 (74%) White, 2915 (17%) Black, 805 (3.2%) Asian, 76 (0.6%) AI/NA, and 834 (5.8%) Multiracial participants. Mean age, sex ratios, and parental support scores were approximately even across racial groups. White and Asian participants’ households had parents with higher educational attainment, higher equivalence-scaled income, and lower neighborhood disadvantage scores compared to Black and AI/NA participants. On these measures, Multiracial participants were less advantaged than White and Asian but more advantaged than Black and AI/NA participants.

**Table 1:** Participant characteristics and outcomes^a^ stratified by race, Add Health 1994–2008

Characteristic	Overall	White	Black	Asian	AI/NA	Multiracial
	12,372 (100%)	7742 (74%)	2915 (17%)	805 (3.2%)	76 (0.6%)	834 (5.8%)
Male sex	5778 (51%)	3672 (51%)	1267 (50%)	418 (53%)	41 (64%)	380 (50%)
Age	29.0	28.9	29.2	29.2	28.8	28.8
Highest parental education						
Less than high school^b^	1187 (11%)	685 (9.6%)	356 (17%)	69 (13%)	13 (29%)	64 (11%)
High school diploma	3883 (35%)	2520 (34%)	959 (42%)	164 (24%)	19 (34%)	221 (33%)
Some college	2221 (18%)	1405 (19%)	500 (16%)	106 (10%)	19 (24%)	191 (22%)
College graduate or greater	4897 (36%)	3039 (38%)	1043 (26%)	445 (53%)	24 (14%)	346 (34%)
Household income^c^	23.5	25.5	14.4	27.2	13.2	21.2
Parental support index (0–5)	3.33	3.35	3.26	3.30	3.38	3.30
Neighborhood disadvantage (0–1)	0.14	0.12	0.23	0.13	0.23	0.15
Elevated (≥4) ACEs^d^	4402 (27%)	3207 (25%)	648 (33%)	260 (21%)	15 (40%)	272 (35%)
MetS	3131 (29%)	1799 (27%)	852 (38%)	221 (27%)	35 (56%)	224 (31%)
Hypertension	6082 (52%)	3775 (51%)	1464 (55%)	390 (46%)	46 (66%)	407 (50%)
Depression	2195 (33%)	1652 (35%)	296 (26%)	53 (17%)	11 (32%)	183 (35%)
Asthma	1867 (15%)	1134 (15%)	453 (15%)	90 (10%)	14 (16%)	176 (24%)
Anxiety	1518 (13%)	1179 (15%)	182 (6.0%)	26 (2.9%)	4 (3.3%)	127 (18%)
Suicidal ideation	845 (7.4%)	527 (7.2%)	188 (7.4%)	48 (6.0%)	8 (15%)	74 (10%)
Drug use	3965 (36%)	3049 (41%)	356 (12%)	197 (25%)	30 (46%)	333 (42%)

Abbreviations: ACE = adverse childhood experience; MetS = metabolic syndrome.

a Counts are unweighted; proportions (for categorical variables) and means (for continuous) are pooled estimates from 20 survey-weighted imputations.

b Includes completed vocational school or GED.

c Equivalence-scaled to adjust for household size.

d ACE components measured variously across Waves 1, 3, and 4, and summed after imputation; see Appendix A for details.

Exposure to elevated ACEs was highest among AI/NA (40%), Multiracial (35%), and Black participants (33%). Multiracial participants had the highest prevalence of asthma (24%) and anxiety (18%), and along with Whites, the highest prevalence of depression (35%). For all other outcomes, AI/NA participants had the highest prevalence. Asian participants reported the lowest prevalence of all outcomes except for drug use, for which Black participants had the lowest prevalence (12%). White and Asian participants had the lowest prevalence of MetS (27%).

Table 2 summarizes within-group RRs and 95% CIs for each outcome. For the overall sample, increased risks associated with elevated ACEs were null for MetS and hypertension, small (4–5%) for asthma, anxiety, and suicidal ideation, and stronger (10–13%) for depression and drug use. Risks of asthma, anxiety, depression, and drug use increased 12–14% among Multiracial participants, and of drug use by 30% for AI/NA participants. Associations were strongest with depression and drug use (8–13% increased risk) for White and Black participants, and with depression, suicidal ideation, and drug use (8–10%) for Asians. We estimated RRs for interaction using Multiracial participants as the referent group; Table 3 summarizes the exponentiated interaction term betas from each model. Values covered by CIs around interaction terms in the asthma model were also consistent with weaker associations for White (0.92, 95% CI: 0.85, 1.00), Black (0.91, 95% CI: 0.83, 1.00) and Asian (0.89, 95% CI: 0.79, 1.00) participants. CIs around interaction terms in the anxiety model were also consistent with stronger association for Multiracial individuals compared to all other groups. CIs for (Black (0.93, 95% CI: 0.86, 0.99), Asian (0.87, 95% CI: 0.82, 0.94), AI/NA (0.89, 95% CI: 0.81, 0.97) participants did not include the null.

**Table 2:** Adjusted^a^ overall and within-group RRs and 95% CIs associated with elevated ACEs, Add Health 1994–2008

Outcome	Overall	White	Black	Asian	AI/NA	Multiracial
MetS	1.00 (0.98, 1.02)	1.03 (0.94, 1.12)	1.00 (0.97, 1.02)	1.00 (0.96, 1.05)	0.99 (0.87, 1.13)	1.00 (0.78, 1.28)
Hypertension	0.99 (0.97, 1.01)	0.99 (0.97, 1.01)	1.01 (0.96, 1.05)	1.02 (0.90, 1.16)	0.86 (0.69, 1.07)	0.98 (0.91, 1.04)
Asthma	1.04 (1.03, 1.06)	1.04 (1.02, 1.06)	1.02 (0.98, 1.07)	1.00 (0.93, 1.08)	1.15 (0.93, 1.42)	1.13 (1.04, 1.22)
Depression	1.13 (1.10, 1.16)	1.13 (1.09, 1.16)	1.13 (1.08, 1.18)	1.10 (0.98, 1.24)	1.21 (0.90, 1.61)	1.14 (1.07, 1.22)
Anxiety	1.05 (1.03, 1.07)	1.05 (1.03, 1.08)	1.03 (1.00, 1.06)	0.98 (0.95, 1.00)	0.99 (0.92, 1.07)	1.12 (1.04, 1.19)
Suicidal ideation	1.05 (1.03, 1.07)	1.05 (1.03, 1.07)	1.05 (1.01, 1.09)	1.08 (1.00, 1.17)	1.07 (0.84, 1.37)	1.05 (0.98, 1.12)
Drug use	1.10 (1.08, 1.13)	1.11 (1.08, 1.13)	1.08 (1.03, 1.12)	1.08 (0.95, 1.23)	1.30 (1.06, 1.60)	1.13 (1.07, 1.20)

Abbreviations: RR = risk ratio, CI = confidence interval, ACE = adverse childhood experience; MetS = metabolic syndrome.

a Models adjusted for participant age, sex, parental education, household size-adjusted income, parental support, and neighborhood disadvantage score.

**Table 3:** Exponentiated interaction^a^ term betas and 95% CIs from regression analyses, Add Health 1994–2008

Outcome	White	Black	Asian	AI/NA	Multiracial
MetS	0.97 (0.89, 1.06)	0.98 (0.89, 1.07)	0.97 (0.82, 1.14)	0.97 (0.76, 1.24)	(ref.)
Hypertension	1.01 (0.94, 1.09)	1.03 (0.95, 1.12)	1.05 (0.91, 1.21)	0.88 (0.70, 1.10)	(ref.)
Asthma	0.92 (0.85, 1.00)	0.91 (0.83, 1.00)	0.89 (0.79, 1.00)	1.02 (0.82, 1.28)	(ref.)
Depression	0.98 (0.91, 1.06)	0.99 (0.91, 1.07)	0.96 (0.84, 1.10)	1.05 (0.78, 1.42)	(ref.)
Anxiety	0.94 (0.88, 1.02)	0.93 (0.86, 0.99)	0.87 (0.82, 0.94)	0.89 (0.81, 0.97)	(ref.)
Suicidal ideation	1.00 (0.93, 1.08)	1.00 (0.92, 1.09)	1.03 (0.93, 1.15)	1.02 (0.79, 1.32)	(ref.)
Drug use	0.98 (0.92, 1.04)	0.95 (0.89, 1.02)	0.96 (0.83, 1.11)	1.15 (0.93, 1.42)	(ref.)

Abbreviations: CI = confidence interval, ACE = adverse childhood experience; MetS = metabolic syndrome.

a Race × elevated ACEs interaction, Multiracial as referent group.

Table 4 summarizes excess cases per 1000 associated with elevated ACEs, with the Multiracial group as the reference. Results were mostly consistent with interaction RRs in Table 3. CIs around estimates in the asthma model suggested fewer excess cases for White (−123, 95% CI: −251, −4), Black (−141, 95% CI: −285, −6), and Asian (−169, 95% CI: −334, −7) participants. There were fewer excess cases of anxiety for Black (−100, 95% CI: −189, −10), Asian (−163, 95% CI: −247, −79), and AI/NA (−144, 95% CI: −252, −42) participants; the estimate and CI for Whites (−71, 95% CI: −165, 25) also suggested a weaker association compared to Multiracial participants. CIs around excess case estimates were wide for some outcomes, especially for the AI/NA and Asian groups.

**Table 4:** Excess^a^ cases^b^ per 1000 and 95% CIs^c^ associated with elevated ACEs, Add Health 1994–2008

Outcome	White	Black	Asian	AI/NA	Multiracial
MetS	−26 (−108, 52)	−20 (−108, 62)	−27 (−176, 117)	−23 (−263, 239)	(ref.)
Hypertension	14 (−65, 96)	33 (−56, 125)	49 (−100, 218)	−148 (−382, 116)	(ref.)
Asthma	−123 (−251, −4)	−141 (−285, −6)	−169 (−334, −7)	23 (−287, 383)	(ref.)
Depression	−17 (−114, 76)	−30 (−133, 67)	−65 (−225, 96)	63 (−278, 494)	(ref.)
Anxiety	−71 (−165, 25)	−100 (−189, −10)	−163 (−247, −79)	−144 (−252, −42)	(ref.)
Suicidal ideation	3 (−83, 87)	1 (−95, 91)	38 (−87, 166)	30 (−271, 354)	(ref.)
Drug use	−50 (−199, 88)	−147 (−309, 2)	−122 (−429, 199)	359 (−140, 865)	(ref.)

Abbreviations: CI = confidence interval, ACE = adverse childhood experience; MetS = metabolic syndrome.

a Multiracial as referent group.

b Rounded to nearest whole person.

c 95% CIs calculated from 10,000 resamples of interaction contrast.

Appendix E summarizes results from the first sensitivity analysis which specified summary ACE score (rather than ≥4 ACEs) as the exposure, keeping Multiracial participants as the reference. Direction and magnitude of results were similar to the main analysis. Appendix F displays results from the second sensitivity analysis which specified White participants as the reference, keeping elevated ACEs as the exposure. Directions of results were consistent with the main analysis.

## 4 Discussion

Our study aimed to explore whether the association of ACEs with physical, mental, and behavioral health outcomes differs by race, and in particular, if Multiracial individuals should be prioritized for interventions given their unique ACEs and health risk profile. We found evidence that, compared to Black, Asian, and AI/NA (but not White) participants, Multiracial people experience greater absolute and relative strength of association with ACEs, regardless of ACEs coding scheme. This finding contrasts with previous research has suggested that ACEs are more strongly associated with count of poor mental health days for Multiracial study participants compared to Whites. However, our measure of anxiety included those taking anxiety medications, which could lead to fewer poor mental health days, making these outcomes potentially less correlated. We also estimated that Multiracial participants have more excess cases of asthma associated with elevated ACEs compared to most other racial groups examined. Our findings also contrasts with results from previous research (Hall et al., 2020) which found no difference in strength of associations between the Multiracial and Asian (referent) groups. However, that study estimated odds ratios, which may be a distorted representation of risk given the high prevalence of asthma in the population.

Data from the National Health Interview Survey show that prevalence of asthma and anxious symptoms are disproportionately and significantly higher among Multiracial people than other racial groups; for asthma, these patterns remain after disaggregation of specific Multiracial sub-groups such as Black-White and AI/NA-White (National Center for Health Statistics, 2018b; National Center for Health Statistics, 2018d). However, there is a dearth of research exploring the various pathways leading to elevated risk of asthma and anxiety among Multiracial people specifically, and thus these disparities remain unexplained. Although ACEs are a well-established risk factor for a variety of poor adulthood health outcomes, the structural and social processes that determine disparities in exposure to ACEs, and the causes of variability in race-specific associations remain poorly understood. As previously discussed, asthma and anxiety are both linked to the biological embedding of trauma through the stress pathway and epigenetic expression, but may also have etiologic origins in further upstream structural factors such as segregation and structural racism (Clausing et al., 2023; Martinez et al., 2021, 2023). While our study does not explore structural processes or the causes of disparities in exposure and associations, it does highlight the contribution of ACEs to asthma and anxiety disparities for Multiracial adults. Future studies should examine proximal factors including ACEs as situated within a network of further upstream causes; such research can help evaluate and compare the relative benefits of potential interventions and their impact on health equity.

Despite Multiracial children's high rate of exposure to ACEs, and even though the 2020 Census reports that one in ten people selected two or more races (Bureau UC, 2020), health equity research largely continues to overlook the Multiracial population. Policies and programmatic interventions are needed to specifically address inequities in asthma and anxiety among the Multiracial population. The Centers for Disease Control's (CDC) strategies for ACEs prevention includes ensuring a strong start for children, promoting skills for parents and children to manage stress and emotions, and connecting children to caring adults and activities (Centers for Disease Control and Prevention, 2019, p. 40; Centers for Disease Control and Prevention, 2021). Practitioners involved in designing and implementing ACEs prevention programs should tailor these strategies to provide supports specific to the social experiences and challenges Multiracial children face. For example, Multiracial youth may experience microaggressions from monoracial peers, family members, and strangers on the basis of their racial identities (Franco & Carter, 2019; Franco et al., 2016; Franco & Franco, 2016; Harris, 2017; Johnston et al., 2010); these challenges to healthy racial identity development can result in risks (e.g. maladaptive behaviors (Franco & Carter, 2019; Goings et al., 2018)) but also development of resilience mechanisms (Yoo et al., 2016) to adapt and thrive in spite of those risks. Promoting community- or school-based “third spaces" (Gabriel et al., 2022; Narvaez & Kivlighan, 2021) – places that allow young Multiracial people to safely explore and discuss their experiences with race among caring peers and adults – could be one example of adapting general CDC strategies to better serve the Multiracial population. Similar peer support programs could also be made available to serve parents of Multiracial children, who may be unfamiliar with the social challenges of growing up as Multiracial in a monocentric society. Finally, while the CDC's strategy of strengthening economic supports to prevent ACEs would be universally beneficial, the downstream health impacts could especially benefit the Multiracial population's mental and respiratory health given the findings of this study and others.

## 5 Limitations

Our study had strengths, including a uniquely large representative and longitudinal sample of Multiracial participants and multiple investigated outcomes. However, there were also limitations. First, the racial categories used in this analysis and health research generally collapse large amounts of within-group variability in patterns of health and disadvantage; subgroup analyses thus produce only a rough proxy for markers of elevated risk and should be contextualized with other knowledge. Second, our ACEs measure is unique to Add Health and had high levels of missingness. Measures for asthma, anxiety, and depression were self-reported, and may be biased downward for racial groups with less healthcare access or where mental health conditions are especially stigmatized (McGuire & Miranda, 2008). Our measure of parental education used highest reported level between either parent rather than primary caregiver, which some research suggests may better predict child health (Braveman et al., 2005). Finally, small sample sizes particularly for Asian and AI/NA groups contributed to wide CIs around excess case estimates; future studies should repeat this analysis in larger longitudinal cohorts with greater numbers of Asian and AI/NA participants.

## 6 Conclusion

To our knowledge, this is the first study finding that exposure to ACEs is associated with excess risk of anxiety and asthma for Multiracial people, a finding that would be obscured if Multiracial people were recategorized into an “other” or monoracial categories. Given the large and growing size of the Multiracial population and inequitable rates of exposure to ACEs among Multiracial children, addressing and preventing ACEs is an urgent health equity issue. While intervening to reduce exposure to ACEs will be universally beneficial for all racial groups, such programs may especially benefit the respiratory and mental health of the Multiracial population. Existing ACEs prevention strategies can also be further tailored to provide targeted supports to young Multiracial people and their parents. Future studies should continue to examine the aggregate and group-specific population health benefits of preventing ACEs to improve health equity.

## Financial disclosures

No financial disclosures were reported by the authors of this paper.

## Funding

TLH was supported by NIH-NCATS-CTSA grant UL1TR003142 and contract 75D30122P12974 with the 10.13039/100000030Centers for Disease Control and Prevention.

## Author statement

**Tracy Lam-Hine**: Conceptualization, Methodology, Software, Formal Analysis, Writing – Original draft preparation. **Corinne Riddell**: Methodology, Formal analysis, Writing – Review & Editing. **Patrick Bradshaw**: Methodology, Formal analysis, Writing – Review & Editing. **Michael Omi**: Conceptualization, Writing – Review & Editing. **Amani Allen**: Conceptualization, Writing – Review & Editing, Supervision.

## Declaration of competing interest

The authors declare no competing interests.

## Data Availability

Restricted data were made available via a data use agreement with the Carolina Population Center. A public use version is available online at https://addhealth.cpc.unc.edu/data/
